# ^1^H-Nuclear Magnetic Resonance Analysis of the Triacylglyceride Composition of Cold-Pressed Oil from *Camellia japonica*

**DOI:** 10.3390/molecules17066716

**Published:** 2012-06-04

**Authors:** Carmen Salinero, Xesús Feás, J. Pedro Mansilla, Julio A. Seijas, M. Pilar Vázquez-Tato, Pilar Vela, María J. Sainz

**Affiliations:** 1 Areeiro Phytopathological Station, Pontevedra Deputation, Subida a la Robleda s/n., E36153 Pontevedra, Spain; Email: carmen.salinero@depo.es (C.S.); pedro.mansilla@depo.es (J.P.M.); pilar.vela@depo.es (P.V.); 2 Department of Organic Chemistry, Faculty of Science, University of Santiago de Compostela, E27002 Lugo, Spain; Email: xesus.feas@usc.es (X.F.); pilar.vazquez.tato@usc.es (M.P.V.-T.); 3 Department of Plant Production, University of Santiago de Compostela, E27002 Lugo, Spain; Email: mj.sainz@usc.es

**Keywords:** *Camellia japonica*, nuclear magnetic resonance, ^1^H-NMR, ^13^C-NMR, triacylglycerides, oil

## Abstract

*Camellia japonica *(CJ) has oil-rich seeds, but the study of these oils has received little attention and has mainly focused only on their health properties. In the present work the relative composition of the fatty acid (FA) components of the triglycerides in cold-pressed oil from CJ is studied by ^1^H-NMR. The results obtained were: 75.75%, 6.0%, 0.17% and 18.67%, for oleic, linoleic, linolenic and saturated FA respectively. Levels of C_18_ unsaturated FA found in CJ oil were similar to those reported for olive oils. We also checked the possibility of using ^13^C-NMR spectroscopy; however, the results confirmed the drawback of ^13^C over ^1^H-NMR for the study of FA components of CJ triglycerides due to its low gyromagnetic ratio and its very low natural abundance.

## 1. Introduction

The genus *Camellia*, comprising more than 200 species, includes evergreen shrubs and trees belonging to the *Theaceae* family, which are grown mainly for the preparation of tea with the leaves and buds, for the seeds in order to obtain oil, and as ornamental plants [1]. Although the *Camellia* is native to Asia [2], the cultivated species are well adapted in several other countries and possess great economic value. Among the camellia species, the economic value of the *Camellia japonica* (CJ) ranks the highest due to its beautiful ornamental flowers. Indeed, over 32,000 cultivars are registered [3]. In Galicia (NW Spain), one of the most important *Camellia* producing-regions in Europe, about 2.5 million *Camellia* plants (most of them CJ) are produced in nurseries for use as houseplants and in gardening each year and then exported to markets in Belgium, The Netherlands, France, the United Kingdom and Portugal.

CJ does not only have an ornamental value, as several parts of the plant, including leaves, bark, flowers, flower buds, twigs, seeds, the pericarp and roots, have been used traditionally in oriental ethnomedicine for health purposes, such as the treatment of stomach disorders, blood vomiting and bleeding due to internal and external injury, as well as a tonic and anti-inflammatory agent [4].

As chemical constituents of this natural medicine, several triterpenes, flavonoids and phenolic compounds were reported [5,6,7,8,9,10,11,12]. Moreover, when analyzing and studying the therapeutic properties of CJ, modern science has made it possible to specify the potential medical significance of their antimicrobial [13], antioxidant [14,15], antitumoral [16], neuronal cell protective [17], antihistaminic and anti-allergic [18,19,20], antiviral [21,22], hypoglycaemic potential [23], ethanol absorption inhibition [24] and skin healing properties [25].

Seeds of several species belonging to the *Oleifera*, *Paracamellia*, *Camellia*, and *Furfuracea* sections are used to extract high quality edible oil [26]. With a unique flavor and taste, good storage stability and claimed health benefits, *Camellia* oil, mainly obtained from wild plants of the species *C. oleifera*, *C. semiserrata*, and *C. chekiangolomy*, is often a target for adulteration or mislabeling in China because it is a high priced product with high nutritional and medicinal values [27,28]. Surprisingly, data on *Camellia* oil in the referred literature is scarce, and mainly focused on *C. oleifera* oil (tea seed oil), since in central and south China it is used extensively as a cooking oil.

Edible oils are mainly made up of triacylglycerols (TAG) which comprise more than 95 to 99% of the total lipids present. In TAG the HO- of the glycerol joins the -COOH of the fatty acid (FA) to form ester bonds. Because there are a large number of individual FAs, with different chain lengths, and degrees of unsaturation and position on the glycerol molecules, defining the TAG composition of an oil is a very challenging task [29]. In fact, each type of oil has a different TAG profile which determines the nature of its physicochemical and nutritional properties, and also provides information on the quality of the oil. In recent years, both industry and consumers have shown an increased interest in the latter.

Our literature survey revealed that several methods for the qualitative and quantitative determination of TAGs in oil samples are available [29,30,31]. These techniques include mainly gas-liquid chromatography, high performance chromatography in normal and reversed phase mode, thin-layer chromatography and supercritical fluid chromatography. However, these methods are labor-intensive and time consuming and also involve a complex series of chemical manipulation steps.

Nuclear magnetic resonance (NMR) has become one of the most promising methods to determine organic structures in complex matrices such as foods, and pharmaceutical and biological samples [32,33]. ^1^H nuclear magnetic resonance (^1^H-NMR) offers many advantages over alternative analytical methods to study edible oils because it allows the rapid, simultaneous, noninvasive, and nondestructive study of oil composition, and also provides information about the acyl distribution and the acyl positional distribution of TAGs [34,35,36,37].

Although Galicia is one of the most important *Camellia japonica* producing-regions in Europe, there is no information available on the composition of CJ oil from this region. This lack of knowledge commonly leads to the waste of this quite easily obtained product. Thus, most of the Galician producers are not able to maximize their profits. In this context, the characterization and standardization of CJ oil would allow for the increase of its use in industries, and as a consequence, the economic value of this natural resource. All of this would stimulate its production. At the same time, it is necessary to measure elemental composition in order to provide correct denominations that can establish the minimum marketing level of the product and provide adequate consumer protection. The aim of this work was to study the TAG composition of cold pressed oil obtained from *C. japonica* cultivated in NW Spain by ^1^H-NMR analysis.

## 2. Results and Discussion

**Figure 1:**
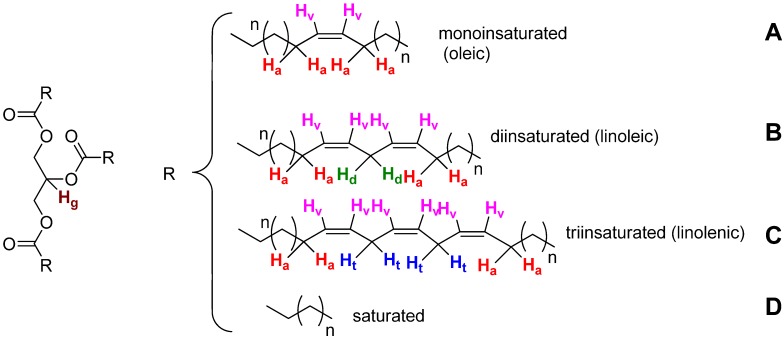
Chemical structure of the main triacylglycerols in oils.

The structure of the major TAGs present in CJ oil highlight the most singular kinds of hydrogen from the NMR point of view, and is represented in Figure 1. Vinylic hydrogens (H_v_) have a characteristic chemical shift, and could be used to determine the ratio of saturated to unsaturated esters. Bisallylic hydrogens (H_d_, H_t_) could be used to differentiate the nature of the unsaturated components. Finally, the tertiary hydrogen in the glycerin moiety (H_g_) could be used to quantify the ratio of saturated to unsaturated esters since there is only one hydrogen for each TAG molecule.

The proton resonances of the TAG acyl chains were assigned according to the literature data [38,39] and are shown in Table 1 and Table 2.

**Table 1:** Assignment of the signals of *Camellia japonica* oil ^1^H-NMR spectra (300 MHz for ^1^H).

Signal	Functional group	Multiplicity	Chemical shift (ppm)
**1**	I (dd) –CH_3_	dd	0.96–0.82
**2**	H (m) –CH_2_–	m	1.43–1.16
**3**	G (m) –CH_2_–C–CO_2_	m	1.70–1.51
**4**	F (m) –CH_2_–CO_2_–	m	2.11–1.91
**5**	E (m) –C–CH_2_–C=C–	m	2.38–2.21
**6**	D (t) –C=C–CH_2_–C=C––C=C–CH_2_–C=C–CH_2_–C=C	t	2.83–2.73
**7**	C (dd) –C–CH_2_–O–CO–C	dd	4.21–4.08
**8**	B (dd) –C–CH_2_–O–CO–C	dd	4.36–4.22
**9**	A (m) CH(–C–O–CO–C–)_2_+ C–HC=CH–C	m	5.43–5.13

**Figure 2:**
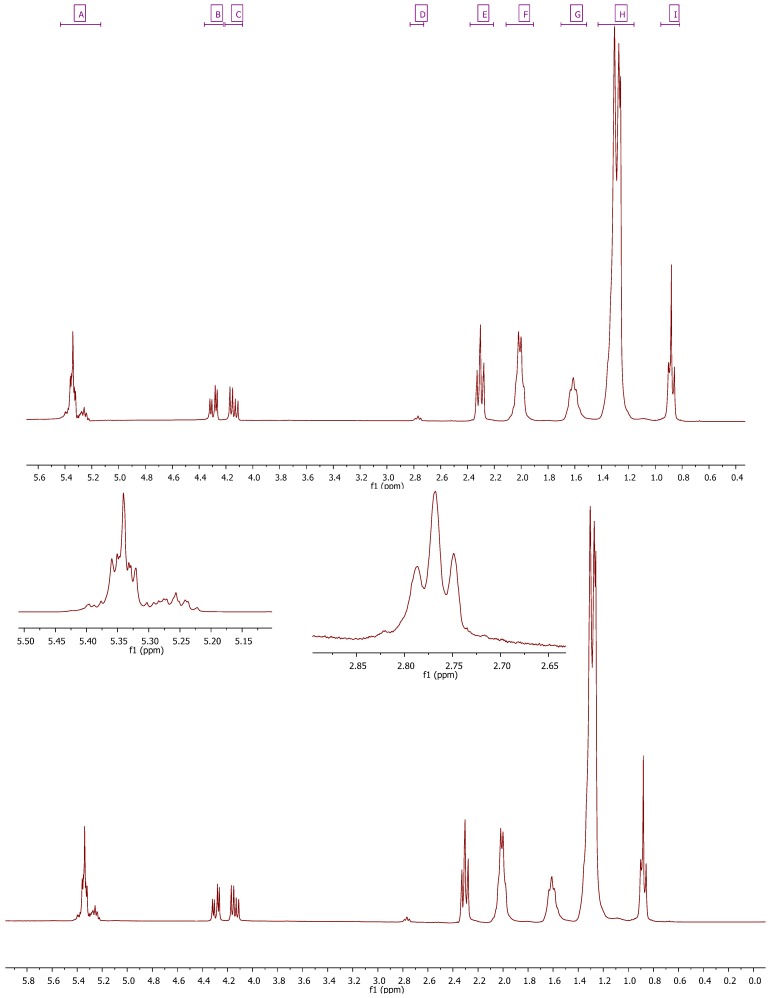
*Camellia japonica* oil ^1^H-NMR spectra (300 MHz for ^1^H).

Signal multiplicity: s, single; d, doublet; t, triplet; m, multiplet. The signal number agrees with those in Figure 2.

**Table 2:** Assignment of the signals of *Camellia japonica* oil ^1^H-NMR spectra (750 MHz for ^1^H).

Signal	Functional group	Multiplicity	Chemical shift (ppm)
**1**	I (t) –CH_3_	t	0.89–0.86
**2**	H (m) –CH_2_–	m	1.35–1.23
**3**	G (m) –CH_2_–C–CO_2_–	m	1.64–1.57
**4**	D (m) –CH_2_–CO_2_–	m	2.02–1.98
**5**	E (m) –CH_2_–CO_2_–	m	2.06–2.02
**6**	F (dt) –C–CH_2_–C=C–	dt	2.33–2.28
**7**	C (t) –C=C–CH_2_–C=C–	t	2.78–2.74
**8**	L (m) –C=C–CH_2_–C=C–CH_2_–C=C	m	2.81–2.78
**9**	A (dd) –C–CH_2_–O–CO–C	dd	4.15–4.06
**10**	B (dd) –C–CH_2_–O–CO–C	dd	4.30–4.26
**11**	K (m) CH(–C–O–CO–C–)_2_	m	5.27–5.24
**12**	J (m) C–HC=CH–C	m	5.37–5.30

**Figure 3:**
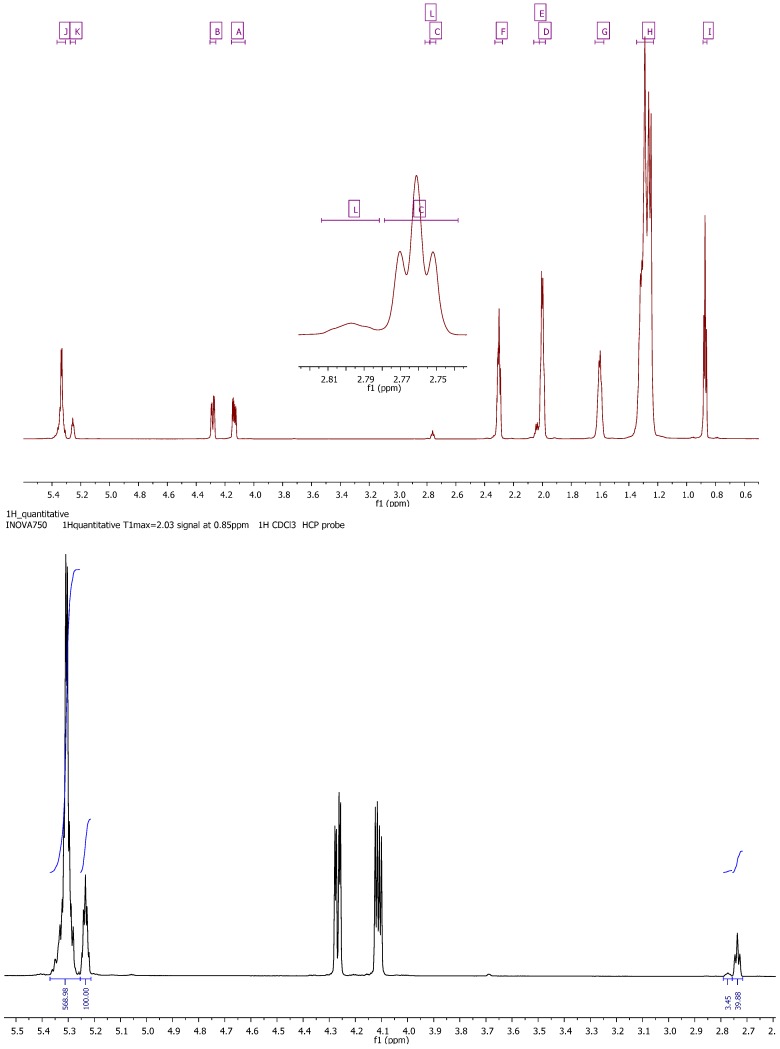
*Camellia japonica* oil ^1^H-NMR spectra (750 MHz for ^1^H).

Signal multiplicity: s, single; d, doublet; t, triplet; m, multiplet; dt, double of triplet; dd, doublet of doblet. The signal number agrees with those in Figure 3.

We studied the 300 MHz spectra for CJ oil (Figure 2); however the spectra obtained did not allow for the accurate integration of the tertiary hydrogen Hg of the glycerine moiety, and no difference was found between bisallylic hydrogens (δ = 2.80 ppm approx.).

The use of a 750 MHz spectrometer to analyze the samples rendered a higher resolution spectra that in turn allowed for the separated integration of the signal from the tertiary glycerin hydrogen, and the vinylic ones (Figure 3). For CJ oil, once the peak of H_g_ was normalized to 100, the signal of vinyl hydrogens was integrated as 5.26 (5.37–5.50 ppm), the bisallylic hydrogens giving 0.36 (linoleic) and 0.02 (linolenic).

The equations were defined as follows, with A, B, C and D as the fractions of each kind of FA involved in the TAG structure:


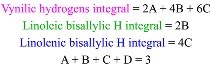
(1)

The measured integrals [40] can be summarized as: H_v_ = 568.98, H_d_ = 39.88, H_t_ = 3.45 and H_g_ = 100. Thus the values of each kind of acid are: A 80.67%, B 6.65%, C 0.29% and D 12.39%, for oleic, linoleic, linolenic, and saturated fatty acids, respectively.

Table 3 shows the typical levels of C_18_ unsaturated and total saturated FA reported in literature for common oils. The values recorded for linoleic, linolenic and oleic FA were similar to those obtained for olive oils.

**Table 3:** Typical levels (in %) of C_18_ unsaturated fatty acids and total saturated fatty acids in common oils.

Oil	Linoleic	Linolelic	Oleic	Saturated
Virgin olive ^a^	6.0	0.7	81.3	12
Hazelnut ^a^	13.5	0.0	77.9	8.6
Peanut ^a^	21.6	0.0	61.8	16.6
Virgin olive ^b^	5.9	0.7	80.0	13.4
Olive ^b^	7.4	0.7	77.5	14.4
Hazelnut ^b^	10.7	0.0	81.0	8.3
Corn ^b^	51.0	0.7	33.0	15.3
Sunflower ^b^	58.8	0.0	29.2	12.0
Soybean ^b^	54.2	10.4	20.4	15.0
Linseed ^b^	17.1	54.2	20.0	8.7
Avocado ^c^	10	1	65	20
Tea seed oil ^c^^,^*	10	<1	80	10
Pumpkin ^c^	40	<1	40	10
Soybean ^c^	50	7	25	15
Canola ^c^	20	10	60	7
Olive ^c^	8	<1	75	14
Camellia japonica **	6.65	0.29	80.67	12.39

^a^ [41]; ^b^ [42]; ^c^ [43]; * Oil from*Camellia oleifera*; ** Present work.

**Figure 4:**
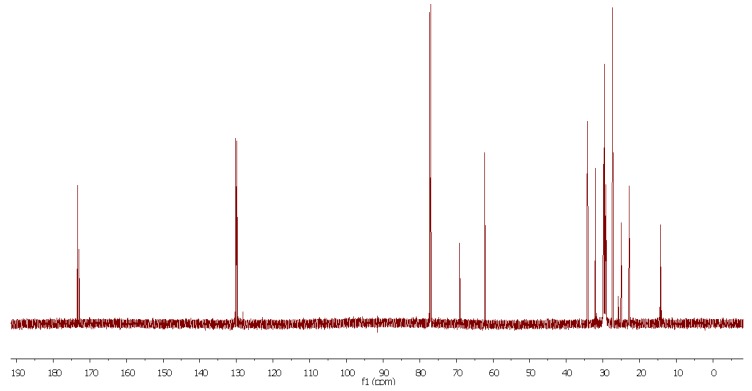
*Camellia japonica* oil ^13^C-NMR spectra (189 MHz).

**Figure 5:**
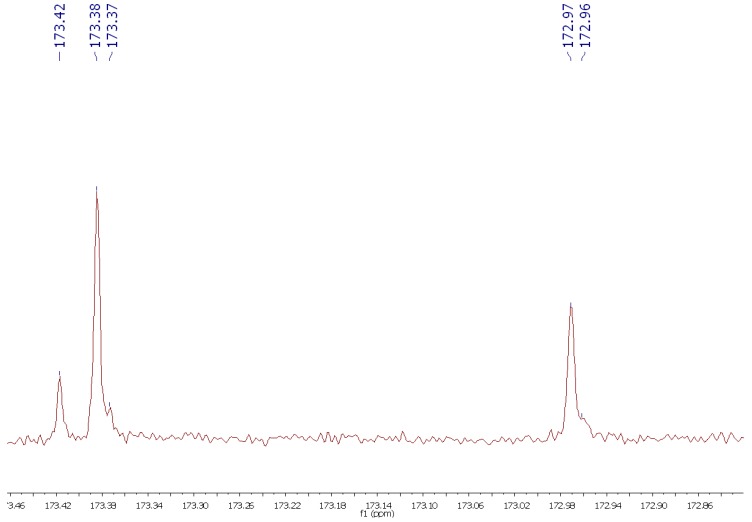
*Camellia japonica* oil ^13^C-NMR spectra (189 MHz) of carbonyl (top) and vynilic (bottom) regions.

We also checked the possibility of using ^13^C-NMR spectroscopy for the rapid analysis of CJ oil [44]. Although this technique has been used for the study of the composition of several oils, its main drawback is the low natural abundance of the ^13^C isotope. The same sample used for the ^1^H-NMR experiment was used for the ^13^C acquisition (40 min, 1107 scans) and Figure 4 shows the spectrum obtained without any apodization. The carbonyl region showed four peaks (Figure 5 top): 173.42 corresponds to the carbonyl group in saturated fatty ester chains, 173.38 and 172.97 to the carbonyls in oleic esters, and 173.37 and a shoulder at 172.96 to linoleic ester chains.

The characteristic vinylic hydrogen region (Figure 5 bottom) showed peaks of oleic ester at 130.16, 130.14, 129.84, and 129.81, together with 130.35, 130.11, 120.20, and 128.02 from linoleic esters, but no peaks corresponding to linolenic could be detected. These results confirmed the well known handicap of ^13^C for rapid analysis of TAGs due to its low gyromagnetic ratio and its very low natural abundance.

## 3. Experimental

### 3.1. Oil Obtention

The extraction of *Camellia japonica* oil (CJO) essentially involved preconditioning of camellia fruits, mechanical pressing of seeds and filtering to remove oil impurities. Mature *Camellia japonica* fruits were obtained from healthy plants grown in the live germplasm camellia bank at the Estación Fitopatolóxica do Areeiro (Pontevedra, Galicia, Spain). The fruits are large apple-shaped woody capsules that can contain several seeds, which are dropped on to the ground in late summer. For the study, ripe fruits were collected as soon as the capsules began to split open, and allowed to dry at room temperature for a week. Then seeds were removed from capsules. After crushing, seeds were cold-pressed for oil. The oil often had suspended particles in it which had to be filtered out. Finally, the CJ oil obtained was stored in amber glass bottles at ambient temperature.

### 4.2. ^1^H-NMR Analysis

^1^H-NMR analyses were performed on Varian Mercury 300 (300 MHz for ^1^H) and Varian Inova 750 (750 MHz for ^1^H) instruments (Agilent Technologies®, Palo Alto, CA, USA), equipped with a 5 mm probe. Each oil sample, weighing 0.2 g, was dissolved in 400 µL of deuterated chloroform CDCl_3_, (Sigma-Aldrich®, Madrid, Spain) shaken in a vortex mixer, and the resulting mixture was placed into a 5-mm diameter ultra-precision NMR sample tubes (Norell®, Landisville, PA, USA). The temperature of the sample in the probe was 30 °C. The chemical shifts are reported in ppm, using the solvent proton signal as standard. The area of the signals was determined by using the equipment software, and the integrations were carried out three times to obtain average values. All figures of the ^1^H-NMR spectra and of the expanded ^1^H-NMR spectrum regions were plotted at a fixed value of absolute intensity to be valid for comparative purposes.

### 4.3. ^13^C-NMR Analysis

^13^C-NMR analysis was performed on a Varian Inova 750 spectrometer working at 189 MHz for ^13^C. The same samples subjected to ^1^H-NMR analyses were used for ^13^C-NMR analysis.

## 4. Conclusions

In summary, 750 MHz ^1^H-NMR spectroscopy has proven to be a useful tool for the direct analysis of the triacylglyceride composition of cold-pressed oil from the *Camellia japonica.*
